# A Drought-Activated Bacterial Symbiont Enhances Legume Resilience Through Coordinated Amino Acid Metabolism

**DOI:** 10.3390/microorganisms14010114

**Published:** 2026-01-05

**Authors:** Susmita Das Nishu, Jee Hyun No, Gui Nam Wee, Tae Kwon Lee

**Affiliations:** Department of Environmental and Energy Engineering, Yonsei University, Wonju 26493, Republic of Korea; susmitanishu@gmail.com (S.D.N.); jh_no@yonsei.ac.kr (J.H.N.); guinam.wee@ip-korea.org (G.N.W.)

**Keywords:** drought-specific symbiosis, branched-chain amino acids, conditional mutualism, plant-microbe interaction, transcriptional reprogramming

## Abstract

Drought stress severely impacts agricultural productivity, yet mechanisms underlying microbial enhancement of plant drought tolerance remain poorly understood. This study investigated whether *Sphingobacterium nripensae* DR205 exhibits drought-specific plant growth promotion through conditional metabolic activation. We combined plant cultivation experiments, genome sequencing, and comparative transcriptomics to evaluate DR205 responses under normal and drought conditions with or without root exudates. DR205 showed minimal growth promotion under normal conditions but enhanced plant biomass by 74–344% specifically under drought stress. Genome analysis revealed complete pathways for both stress tolerance (osmolyte biosynthesis and antioxidant systems) and plant interaction (IAA production and nutrient mobilization). Transcriptomics uncovered dramatic metabolic reprogramming under drought, with branched-chain amino acid (BCAA) biosynthesis genes shifting from 27-fold suppression under root exudates to 17-fold upregulation under drought. Lysine biosynthesis showed similar drought-specific activation patterns. Critically, drought signals overrode plant signals maintaining BCAA activation regardless of root exudate presence and ensuring metabolic investment in plant support occurred specifically during water deficit. This conditional mutualism represents a novel bacterial strategy where plant support is selectively activated during environmental stress. These findings challenge conventional PGPR paradigms and offer new approaches for developing climate-resilient agricultural systems through targeted application of stress-responsive beneficial microbes.

## 1. Introduction

Drought stress represents one of the most severe threats to global agricultural productivity, with climate change models predicting increased frequency and severity of water deficit events worldwide [1]. Plants have evolved various physiological and biochemical mechanisms to cope with water shortage, including changes in root architecture, stomatal regulation, osmolyte accumulation, and alterations in root exudate composition [2]. However, plant survival and productivity under drought increasingly depend on their associated microbiomes as beneficial rhizobacteria can enhance plant water stress tolerance through multiple mechanisms, including phytohormone modulation, osmolyte provision, and improved nutrient acquisition [3,4]. While numerous studies have identified plant growth-promoting rhizobacteria (PGPR) that alleviate drought stress, most demonstrate either constitutive beneficial activity that persists regardless of environmental conditions or reduced functionality under stress [5,6]. This conventional understanding of PGPR as constant helpers may not fully capture the complexity of plant–microbe interactions in fluctuating environments where stress occurs periodically rather than continuously.

Branched-chain amino acids (BCAAs), including leucine, isoleucine, and valine, play crucial but underappreciated roles in both plant and microbial drought stress responses. In plants, BCAAs serve as osmolytes for cellular protection, precursors for stress hormones such as jasmonic acid (JA), and alternative respiratory substrates during energy limitation [7,8,9]. Drought stress typically increases plant demand for BCAAs to support enhanced protein turnover and stress-responsive protein synthesis yet simultaneously impairs their biosynthesis due to reduced enzyme activity and cofactor availability [10]. In bacteria, BCAAs contribute to osmotic stress tolerance through synthesis of branched-chain fatty acids that stabilize membranes, production of compatible solutes, and provision of alternative carbon sources for energy metabolism under stress [11,12]. Despite the established importance of BCAAs in stress responses, the possibility that rhizobacteria might specifically upregulate BCAA biosynthesis during drought to support plant partners has not been explored. Furthermore, whether such metabolic support can be selectively activated only under drought conditions, representing an environmentally responsive mutualism, remains unknown.

The current understanding of PGPR functionality is limited by several critical knowledge gaps that prevent full exploitation of plant–microbe interactions for stress resilience. First, most studies evaluate PGPR performance under either optimal or stressed conditions separately, missing potential condition-dependent behaviors [5]. Second, the dynamic nature of microbial metabolic responses to combined environmental and plant signals has been largely overlooked, with most research focusing on static snapshots of gene expression or metabolite production. Third, the concept of conditional mutualism, where beneficial traits are expressed only when environmentally advantageous, has been well-established in mycorrhizal and legume–rhizobia symbioses but remains unexplored in PGPR interactions [5]. These gaps are particularly problematic given that natural environments present fluctuating rather than constant stress conditions, suggesting that bacteria with the ability to modulate their beneficial functions based on environmental cues would have selective advantages. Understanding whether and how PGPR can switch between non-beneficial and beneficial states in response to drought could reveal new strategies for enhancing crop resilience.

The present study investigated *Sphingobacterium nripensae* DR205, isolated from drought-affected soybean rhizosphere soil in Gangwon Province, South Korea, to address these fundamental questions about conditional plant–microbe interactions. We hypothesized that some rhizobacteria have evolved mechanisms to selectively activate plant growth promotion specifically during drought stress, optimizing resource allocation between free-living and symbiotic lifestyles. Our objectives were to (i) determine whether DR205 exhibits differential plant growth-promoting effects under normal versus drought conditions in legume hosts, (ii) characterize the genomic potential and transcriptomic responses underlying any condition-specific behaviors, and (iii) identify key metabolic pathways, particularly focusing on amino acid metabolism, that mediate drought-activated symbiosis. Through integrated physiological, genomic, and transcriptomic approaches, this study reveals a novel form of bacterial–plant interaction where beneficial functions are specifically triggered by environmental stress, challenging conventional paradigms of constitutive PGPR activity and suggesting new insights for developing climate-smart agricultural technologies.

## 2. Materials and Methods

### 2.1. Isolation of the Metabolically Active Drought-Tolerant Strain

Soybean plants were uprooted to collect rhizosphere soil from a temporary and recurrently dry agricultural field (36°58′12.0″ N 128°09′00.0″ E) in Gangwon Province, South Korea. Plants were placed in sterile plastic bags and immediately taken to the laboratory for storage at 4 °C. Bulk soil was gently shaken from the plants, and rhizosphere soil was collected by rinsing the roots in sterile distilled water [13]. The soil suspension was stirred vigorously for 20 min before centrifugation at 8000 rpm for 10 min, and 20 µL of diluted aliquots was plated on tryptic soy agar (BD Difco, Sigma-Aldrich, St. Louis, MO, USA). We isolated the strain by evaluating drought resistance by growing in tryptic soy broth (TSB) (BD Difco, Sigma-Aldrich, St. Louis, MO, USA) supplemented with 25% polyethylene glycol (PEG 6000) (Sigma-Aldrich, St. Louis, MO, USA), which provides an osmotic potential of −1.25 Mega Pascal (MPa) [14,15]. To evaluate the potential of drought tolerance, the isolate was cultured in TSB media amended with and without PEG for 24 h, with 120 rpm at 28 ± 2 °C. Optical density (OD600) was measured using a Spark 10 M spectrophotometer (TECAN, Zurich, Switzerland). Raman spectroscopy was used to study the metabolic activity of the strain under drought (−1.25 MPa) using 40% heavy water (D_2_O) as previously described [16].

### 2.2. Plant Cultivation Experiment

We evaluate the drought alleviation capacity of DR205 by pot test using direct inoculation in legume phenotypes (*Pisum sativum* and *Phaseolus vulgaris*). Bacterial culture (2 mL of 1 × 10^9^ CFU/mL) was injected into each unit of plug tray consisting of 15 g sterilized soil (*Tobirang*, Baekkwang Fertility, Seoul, Republic of Korea) with properties such as pH 6.8, 17.2% moisture, soil texture (10.8% clay, 6.6% silt, and 82.6% sand), total nitrogen content of 3.4 ± 0.2 mg/L, total phosphate content of 1.4 ± 0.1 mg/L, total organic carbon content of 46.7 mg/L, and dissolved organic carbon content of 37.8 mg/L. One pre-germinated seed was sown per unit of the plug tray containing soil properly mixed with bacterial culture. Plug trays were maintained in a 14/10 h light/dark cycle at 30/14 °C with 50% relative humidity, and light intensity was maintained at 1250/0 lux using a growth chamber [17]. In experimental research, the most basic method for inducing drought stress, which represents a soil water deficit condition, is passive pot drying achieved by withholding irrigation [18]. Our preliminary study revealed that withholding watering for seven days reduced the soil water content by approximately 50% compared to well-watered plants under the described growth chamber conditions, which significantly suppressed growth (shoot length, root length, fresh weight, and dry weight) of *P. sativum* and *P. vulgaris*. Full watering was performed once every alternate day for the first ten days, and then drought stress was imposed in the next seven days by halt watering. After seven days of drought stress, plants were re-watered for seven days and harvested. Plants that underwent seven days of drought were considered plants with drought treatment, whereas plants maintained in well-watered conditions were considered plants with non-drought treatment. Plants without inoculated strain DR205 were used as controls for both drought and non-drought conditions. Following the period of rewatering, the soil was gently washed away from the plant roots under low-flowing tap water, and the shoots and roots were separated. We measured shoot length, root length, and fresh and dry root weights. After drying for 24 h at 80 °C, dry root weight was calculated. The plant cultivation experiment was performed with ten biological replicates per treatment (n = 10). Each biological replicate consisted of one plant grown in an individual pot unit. Increase rate was calculated by comparing the growth variables with bacterial treatment to control under both drought and non-drought conditions.

### 2.3. De Novo Sequencing of the Whole Genome

We extracted and purified genomic DNA using FastDNA SPIN KIT (MP Biomedicals, Solon, OH, USA) before sequencing at Macrogen facility (Seoul, Republic of Korea). The Agilent Technologies 2100 Bioanalyzer (Agilent Technologies Inc., Santa Clara, CA, USA) confirmed the quality of the DNA by measuring the size, purity, and concentration of the sample. Genome sequencing was performed using the PacBio RS II system (Pacific Biosciences Inc., Menlo Park, CA, USA) and Illumina HiSeq 2500 (Illumina, San Diego, CA, USA). Sequence reads were assembled using the de novo assembler HGAP3, while contigs were polished using Pilon (v1.21). CheckM version 1.0.0 was used to check for completeness and contamination of the genome. The whole genome sequence of strain DR205 has been deposited in the National Center for Biotechnology Information (NCBI) GenBank under accession number CP048408. The taxonomic identification of strain DR205 was performed using 16S rRNA sequence extraction from the whole genome sequence [19]. The sequence was aligned using the Bioedit program and the phylogenetic tree was constructed using the neighbor-joining method with Molecular Evolutionary Genetics Analysis (MEGA) software version 7.0 [20].

### 2.4. Genome Annotation and Analysis

Genome annotation was performed using the NCBI Prokaryotic Genome Annotation Pipeline with the accuracy of GeneMarkS 2 (v4.7) [21]. Searches for related proteins were performed using the NCBI BLASTp (ver. 2.14) with a cut-off of ≥98% amino acid identity and an *E*-value < 1 × 10^−50^. The whole genome sequence was submitted to Integrated Microbial Genome (IMG) database of the Joint Genome Institute (JGI). The Average Nucleotide Identity (ANI) Calculator of EZBiocloud with the threshold range (95–96%) for species was used to calculate the ANI score between the genome sequence of DR205 and other plant growth-promoting *Sphingobacterium* strains [22]. The presence of gene clusters related to siderophore, secondary metabolites, and EPS was detected using antiSMASH v.5.0 [23].

### 2.5. Evaluating Plant Growth-Promoting Features in DR205 by Biochemical Assay

Indole-3-acetic acid (IAA) production by DR205 was measured by incubating DR205 in 5 mL (104 CFU/mL) Yeast Extract Mannitol (YEM) medium (BD Difco, Sigma-Aldrich, St. Louis, MO, USA) supplemented with 0.1% tryptophan at 120 rpm for 5 days. The bacterial culture (1 mL) was centrifuged at 10,000 rpm for 10 min. The supernatant was treated with Salkowski reagent (2 mL 0.5 M FeCl3, 49 mL 70% perchloric acid, and 49 mL water) at 1:2 for 15 min. OD530 nm was measured using a Spark 10 M spectrophotometer (TECAN, Zurich, Switzerland). IAA production assays were performed with three biological replicates (independent bacterial cultures), each measured in triplicate (three technical replicates).

### 2.6. RNA Extraction, Sequencing, and Differential Expression Analysis

The strain DR205 was cultured in M9 minimal medium [24] at 120 rpm at 28  ±  2 °C for overnight growth. The bacterial culture (1 × 10^9^ CFU/mL) was incubated with three treatments: (i) root exudate (RE), (ii) 25% PEG 6000 (PEG), and (iii) RE + PEG (25% PEG 6000). Synthetic root exudates (REs) were prepared artificially following the composition described in Jin et al. (2019) [25], which represents major components of legume root exudates. The synthetic RE contained sugars (4.65 mM glucose, 1.81 mM arabinose, and 1.54 mM fructose), amino acids (0.31 mM glutamic acid, 0.27 mM aspartic acid, 0.26 mM alanine, and 0.16 mM glycine), and organic acids (0.58 mM fumaric acid, 0.30 mM oxaloacetic acid, 0.07 mM citric acid, and 0.05 mM hydroxamic acid). All components were dissolved in distilled water and sterilized by autoclaving at 121 °C and 15 psi for 20 min. These treatments represent the effects of three conditions: (i) RE mimics plant symbiosis, (ii) PEG mimics drought stress, and (iii) RE + PEG mimics the combination of drought stress and plant symbiosis. Cells cultured in M9 medium with no treatment were considered controls.

RNA extraction was performed using bacteria protect-RNeasy kit (QIAGEN, Hilden, Germany). The purity and concentration of RNA were determined using NanoDrop 2000 (Thermo Fisher Scientific, Wilmington, DE, USA). Agilent Technologies 2100 Bioanalyzer (Agilent Technologies, Santa Clara, CA, USA) was used to measure RNA integrity. Libraries were generated using the Ribo-Zero rRNA Removal Kit (Bacteria) (Epicenter Inc., Madison, WI, USA) and the TruSeq Standard Total RNA Prep Kit (Illumina, San Diego, CA, USA). NovaSeq 6000 System (Illumina-USA) sequencing was performed according to the user manual (Document #1000000019358 v02) by Macrogen (Seoul, Republic of Korea). Sequencing generated an average of 24.3 million reads per sample (range: 20.7–32.4 million reads), providing sufficient depth for differential expression analysis. Quality-filtered reads were aligned to the *S. nripensae* DR205 reference genome (GenBank accession CP049333) using Bowtie2 with default parameters. Mapping rates ranged from 76.1% to 82.4% (average: 78.8%), with all samples achieving Q20 > 98% and Q30 > 94%, confirming high sequencing quality. Detailed sequencing statistics are provided in Table S1. Differentially expressed gene (DEG) analysis was performed on a comparison pair (three treatments vs. control) using reads per kilobase million (RPKM). The significant DEGs represent the genes with fold change |FC| ≥ 2 and *p*-value < 0.05 between the comparison pair, performed by R package “edgeR” (ver. 3.43.3) [26]. Functional annotation based on Kyoto Encyclopedia of Genes and Genomes (KEGG) pathway and Gene Ontology (GO) for possible functional assignment of the genes was performed with protein families of known functions after comparison using the BLASTx program (ver. 2.14) with a threshold of *p* ≤ 0.05 [27].

### 2.7. Statistical Analysis

All statistical analyses used in this study were performed using R software (version 3.4.0) [28]. For plant cultivation experiments, the Shapiro–Wilk test confirmed normal distribution of data (*p* > 0.05). Two-sample *t*-tests were used to evaluate statistical differences between treatment groups, with significance set at *p* ≤ 0.05 and 95% bias-corrected confidence intervals calculated using bootstrap methods (10,000 iterations). Effect sizes were quantified using Cohen’s d. For RNA-seq data, differential expression analysis was performed using edgeR with false discovery rate (FDR) correction, considering genes with |FC| ≥ 2 and FDR-adjusted *p* < 0.05 as significantly differentially expressed.

### 2.8. Repositories

The strain has been deposited to the Korean Culture Center of Microorganisms (accession no. KCCM 43363). The whole-genome sequence of *S. nripensae* DR205 has been deposited to the National Center for Biotechnology Information (NCBI) GenBank under accession number CP049333.

## 3. Results

### 3.1. S. nripensae DR205 Maintains Metabolic Activity Under Severe Drought Stress

To evaluate the drought tolerance capability of *S. nripensae* DR205, we first characterized its plant growth-promoting traits through in vitro biochemical assays. The strain demonstrated substantial production of IAA at 72.91 μg mL^−1^, phosphate solubilization capacity of 397.3 mg L^−1^, and positive siderophore production. These characteristics indicate that DR205 possesses the fundamental biochemical machinery for plant growth promotion, including phytohormone production and nutrient mobilization capabilities.

**Figure 1:**
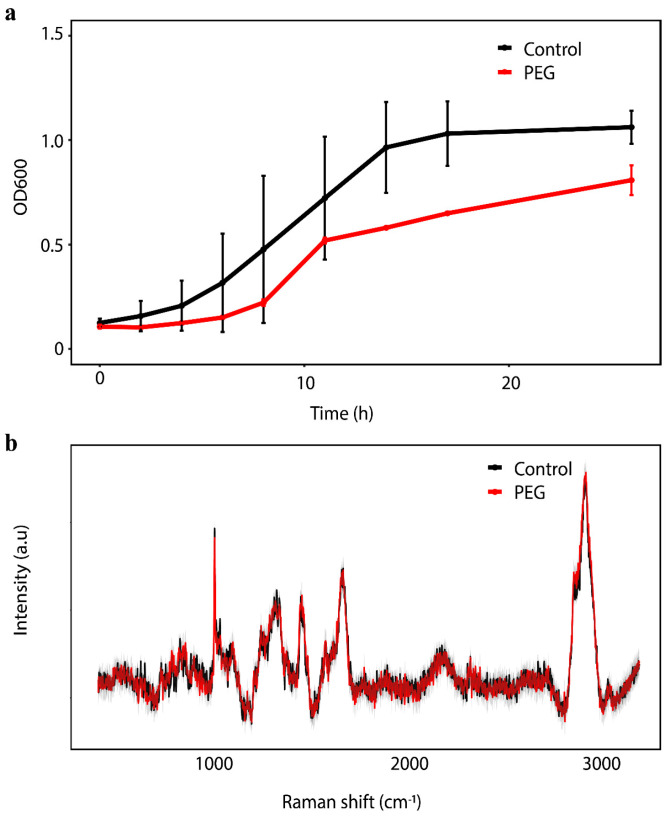
Growth and metabolic activity of strain DR205. (**a**) Growth curve of strain DR205 with 0% and 25% polyethylene glycol (PEG; PEG6000) (−1.25 Mpa). Error bars represent standard deviation from three biological replicates. (**b**) Single-cell Raman spectra (SCRS) of DR205 after 24 h of incubation in medium with 0% and 25% PEG, including 40% deuterium water. Black and red lines represent control and PEG treatments, respectively.

We next examined the strain’s growth and metabolic activity under osmotic stress conditions to determine whether these beneficial traits could be maintained during drought. When cultured in TSB medium supplemented with 25% PEG 6000 (equivalent to −1.25 MPa osmotic potential), DR205 exhibited a notably slower growth rate compared to control conditions (Figure 1a). Despite this growth reduction, the strain maintained continuous growth for 24 h, demonstrating its ability to survive and proliferate under severe water deficit conditions.

To assess whether the reduced growth under drought stress was accompanied by metabolic dormancy or if the cells remained metabolically active, we employed single-cell Raman spectroscopy with deuterium labeling. After 24 h of incubation in medium containing 40% D_2_O, both control and PEG-treated cells showed distinct C-D peaks in the 2040–2300 cm^−1^ region of their Raman spectra (Figure 1b). The presence of these C-D peaks indicates active incorporation of deuterium into cellular biomass, confirming that DR205 maintains metabolic activity even under severe drought stress.

### 3.2. Drought Unlocks Hidden Plant Growth-Promoting Potential

**Figure 2:**
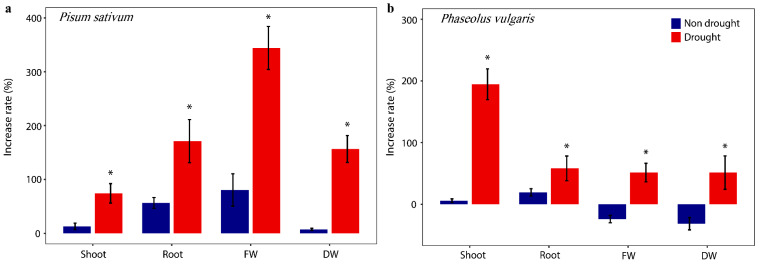
DR205 induced drought tolerance in legume cultivars evaluated by plant cultivation experiment. Effects of inoculation with strain DR205 on plant growth parameters (Shoot: shoot length, Root: root length, FW: fresh weight, DW: dry weight) in (**a**) *Pisum sativum* and (**b**) *Phaseolus vulgaris*. The percentage increase of a growth variable was calculated by the growth under bacterial treatment compared to the non-inoculated control. Blue and red colors indicate plant growth under non-drought and drought conditions. The asterisk indicates a significant difference (*p* < 0.05).

To investigate whether the drought tolerance and plant growth-promoting traits of DR205 translate into actual plant benefits, we conducted pot experiments with two legume species under both well-watered and drought conditions. Remarkably, DR205 inoculation showed contrasting effects depending on water availability, revealing a drought-specific growth promotion pattern. Under well-watered conditions, DR205 inoculation produced moderate improvements in *Pisum sativum*, with increases of 13.1% in shoot length, 56.5% in root length, 80.6% in fresh weight, and 7.5% in dry weight compared to non-inoculated controls (Figure 2a). In *Phaseolus vulgaris*, the effects were even more limited, showing negligible improvements in shoot and root lengths under normal irrigation (Figure 2b). However, under drought stress conditions, DR205 inoculation dramatically enhanced plant growth parameters in both legume species. *Pisum sativum*, drought-stressed plants inoculated with DR205, showed remarkable increases of 74.2% in shoot length, 171.2% in root length, 344.2% in fresh weight, and 156.5% in dry weight compared to drought-stressed controls (Figure 2a).

The effect was particularly pronounced for root development and biomass accumulation, with the fresh weight increasing by more than three-fold. Similarly, *Phaseolus vulgaris* exhibited substantial growth enhancement under drought with increases of 194.6% in shoot length, 48.2% in root length, and 51.3% in both fresh and dry weights (Figure 2b). The differential response between drought and non-drought conditions was statistically significant for all measured parameters (*t*-test, *p* < 0.05, Cohen’s d = 0.45–0.63). Notably, the magnitude of growth promotion under drought exceeded that under well-watered conditions by 2- to 10-fold depending on the parameter measured. This selective enhancement, specifically under water deficit conditions, suggests that the symbiotic interaction between DR205 and plants is triggered or amplified by drought stress signals.

### 3.3. Dual Genomic Capacity Links Stress Tolerance with Plant Interaction

**Figure 3:**
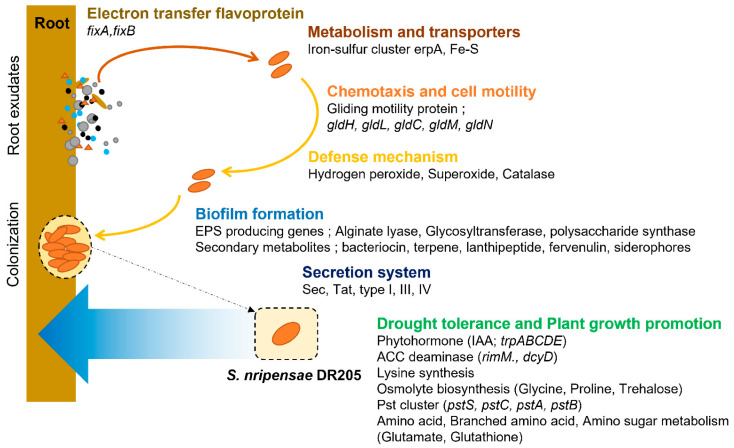
Schematic diagram of functional pathways and genes present in *S. nripensae* DR205. DR205 genome carries gene clusters for electron transfer flavoprotein, chemotaxis and cell motility, antioxidant-based defense mechanisms, EPS and secondary metabolite production, secretion system, phytohormone (IAA), ACC deaminase, osmolyte synthesis, phosphate accumulation, metabolism of amino acid, amino sugar, and branched-chain amino acid.

To understand the genetic basis underlying drought-specific plant growth promotion of DR205, we sequenced and analyzed its complete genome. The genome consists of a single circular chromosome of 7,251,189 bp with a GC content of 40.5%, containing 5956 coding sequences (Figure S1a and Table S2). Phylogenetic analysis based on 16S rRNA sequences confirmed its taxonomic position as *Sphingobacterium nripensae*, while average nucleotide identity (ANI) analysis revealed notably low similarity (78.5%) with the closest sequenced relative, *S. pakistanensis* B29, suggesting that DR205 represents a distinct genomic variant within the genus (Figure S1b). Functional annotation revealed that DR205’s genome harbors extensive genetic machinery for both stress tolerance and plant interaction, organized into distinct but interconnected functional modules (Figure 3).

**Figure 4:**
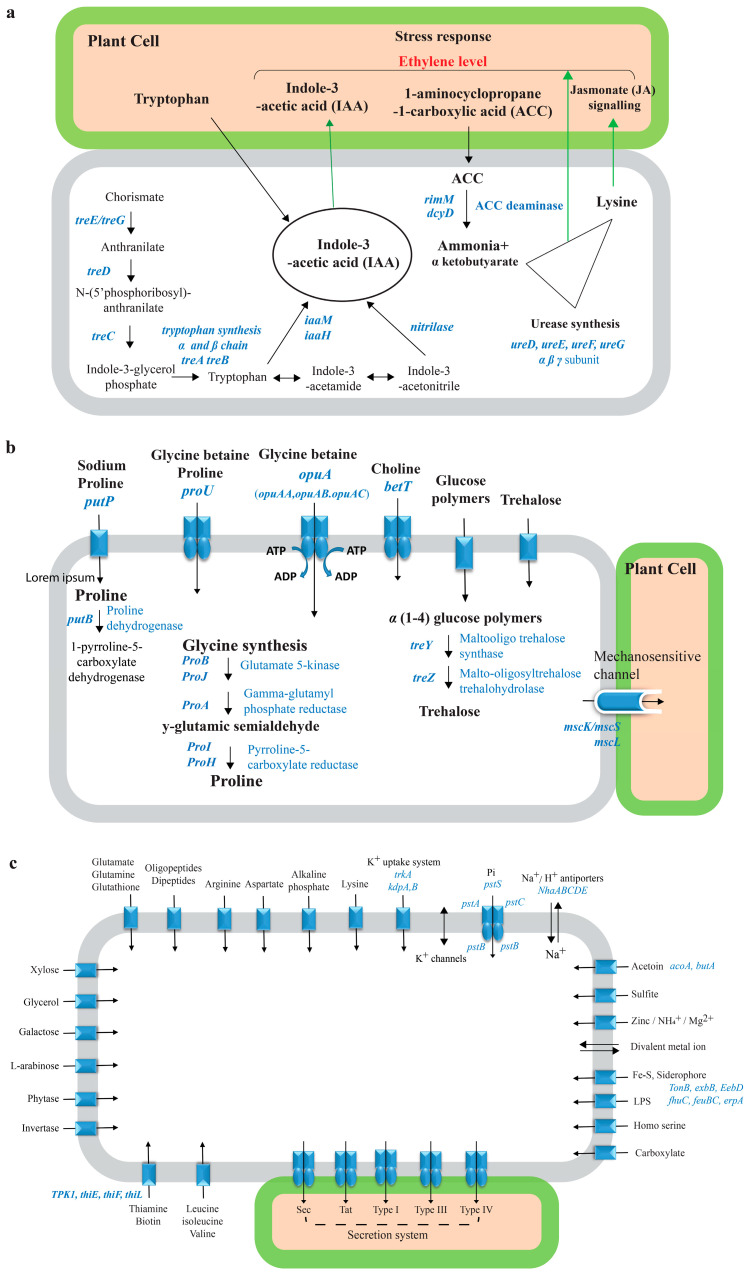
Metabolic pathways and transport systems in the genome of *S. nripensae* DR205. (**a**) Gene clusters and functional pathways involved in the biosynthesis of indole-3-acetic acid (IAA), ACC deaminase, lysine, and urease. (**b**) Biosynthesis and uptake gene clusters for compatible solutes including glycine betaine, choline, proline, glutamate, and trehalose in *S. nripensae* DR205. (**c**) Gene clusters and functional pathways related to transport and exchange of nutrients, including ions (sodium and potassium), phosphate, and structural secretion system gene clusters in DR205.

For drought stress adaptation, we identified complete gene clusters encoding multiple osmolyte biosynthesis and transport systems. These include high-affinity ABC-type glycine betaine transporters (*opuAA*, *opuAB*, and *opuAC*), proline biosynthesis genes (*proB/J*, *proA*, and *proI/H*), and trehalose synthesis pathways (*treY*, *treZ*, and trehalose-6-phosphate synthase). Additionally, the genome contains mechanosensitive channel genes (*mscS*/*mscK*, *mscM*, and *mscL*) that facilitate osmotic adjustment through rapid solute exchange (Figure 4b). The presence of multiple antioxidant defense genes, including catalase, peroxidases, thioredoxin, and superoxide dismutase further equips DR205 to cope with oxidative stress commonly associated with drought conditions.

For plant growth promotion and root colonization, DR205 possesses comprehensive genetic repertoires including the complete tryptophan biosynthesis cluster (*trpABCDE*) for IAA production, ACC deaminase genes (*rimM* and *dcyD*) for ethylene stress mitigation, and urease synthesis genes (*ureDEFG*) for nitrogen supply (Figure 4a). The genome also encodes extensive nutrient acquisition and transport systems, including the high-affinity phosphate transport system (*pstSCAB*), siderophore biosynthesis and transport genes (*tonB*, *exbB*, *exbD*, *fhuC*, and *feuBC*), and multiple carbohydrate utilization pathways for root exudate metabolism (Figure 4c).

Particularly noteworthy is the presence of genes supporting root colonization and plant–microbe signaling. These include extracellular polysaccharide biosynthesis genes for biofilm formation (alginate lyase, glycosyltransferase, and polysaccharide synthase), gliding motility genes (*gldHLCMN*) for root surface movement, and multiple secretion systems (Sec, Tat, Type I, III, and IV) for effector delivery. AntiSMASH analysis identified seven secondary metabolite gene clusters producing bacteriocin, terpene, lanthipeptide, fervenulin, and siderophores that may facilitate competitive root colonization (Table S3).

The genomic architecture of DR205 reveals a complex integration of stress tolerance and plant interaction capabilities, with 8.6% of genes dedicated to transcriptional regulation (COG category K), suggesting complex regulatory networks controlling these dual functions. This unique combination of drought survival mechanisms and plant growth-promoting features provides the genetic foundation for the selective symbiotic behavior of DR205 under water deficit conditions.

### 3.4. Transcriptional Evidence for Drought-Activated Metabolic Specialization

To elucidate how DR205’s genomic potential translates into drought-specific functions, we performed comparative transcriptome analysis under three treatment conditions: root exudates alone, drought stress with 25% PEG (PEG), and combined drought plus root exudates (PEG + root exudate). While the total number of DEGs was similar across treatments, hierarchical clustering revealed that the PEG and PEG + RE samples grouped tightly together and were distinctly separated from the RE treatment, indicating that drought stress dominates the transcriptional response regardless of root exudate presence (Figure S2).

**Figure 5:**
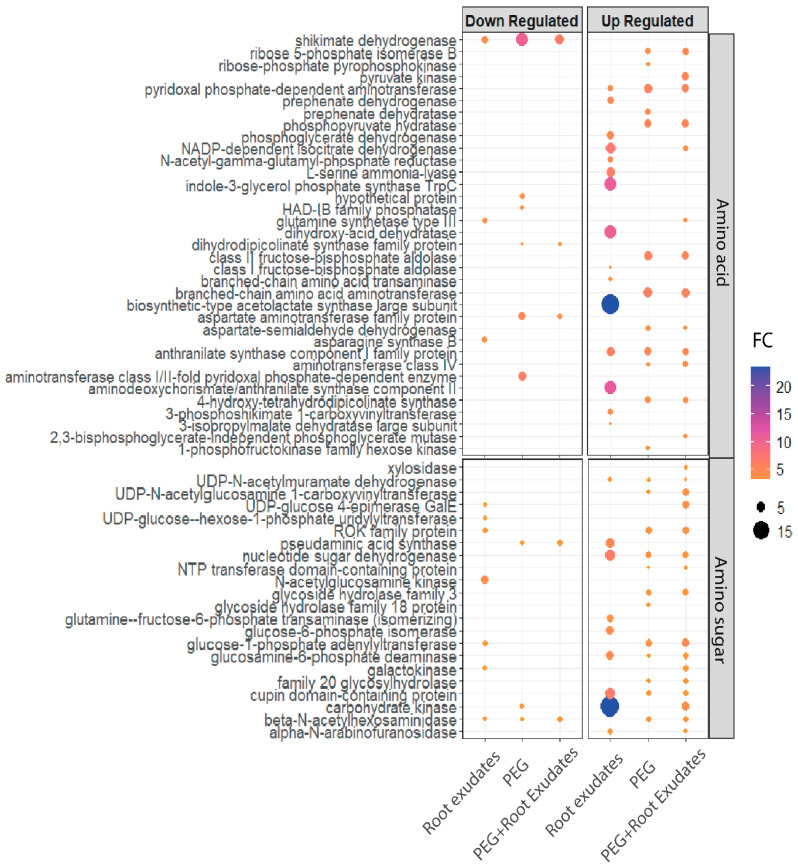
Gene expression of biosynthesis and metabolism of amino acids and amino sugar in *S. nripensae* DR205. Fold change of top up- and downregulated gene clusters related to biosynthesis and metabolism of amino acids and amino sugar in DR205 under the treatments involving root exudates, PEG, and PEG + root exudates. Fold changes represent gene expression in each treatment (root exudates, PEG, and PEG + root exudates) relative to untreated control cells cultured in M9 medium.

Global transcriptional analysis revealed a profound metabolic reorganization under drought conditions. Genes involved in amino acid and amino sugar biosynthesis and metabolism showed widespread upregulation across all treatments, but with markedly different patterns and magnitudes (Figure 5). Under root exudates alone, 24 genes were upregulated with fold changes ranging dramatically from 3- to 24-fold, suggesting a heterogeneous response to plant signals. In contrast, PEG-containing conditions (both PEG and PEG + RE) induced more uniform upregulation of 24 and 29 genes, respectively, with consistent two- to six-fold increases. Notably, only 6 genes were commonly upregulated across all three conditions, while 20 genes showed specific upregulation in PEG-containing treatments, indicating a drought-specific metabolic program.

Analysis of specific metabolic pathways revealed condition-dependent expression patterns. Genes encoding key enzymes in amino acid metabolism, including aminotransferases, dehydrogenases, and synthetases, showed consistent three- to eight-fold upregulation across drought treatments. The amino sugar metabolism pathway, particularly genes involved in glucosamine and N-acetylglucosamine biosynthesis, displayed four- to six-fold increases specifically under PEG treatment, suggesting enhanced cell wall remodeling during osmotic stress.

Interestingly, genes involved in IAA biosynthesis (*trp*A and *trp*B) displayed condition-specific expression patterns. Under root exudates alone, these genes showed robust upregulation of 10- to 18-fold, suggesting active phytohormone production in response to plant signals. However, in PEG-containing conditions, their expression was markedly reduced to only two- to three-fold increases, indicating that drought stress suppresses constitutive phytohormone production despite the presence of plant signals.

The secretion system and quorum-sensing genes maintained relatively stable expression across all conditions (Figure S3a), with only modest changes in preprotein translocase subunits. *Sec*Y showed 14-fold upregulation with root exudates but only a 2-fold increase under drought, while *Yaj*C increased by 7- to 8-fold with root exudates versus 13-fold with PEG alone, suggesting differential regulation of protein secretion pathways under different environmental cues. This transcriptional evidence demonstrates that drought triggers a comprehensive metabolic specialization in DR205, shifting from primary metabolism toward specialized amino acid and stress-related compound biosynthesis, while simultaneously modulating plant interaction pathways in a context-dependent manner.

### 3.5. BCAA and Lysine Biosynthesis Pathways Emerge as Key Mediators of Drought-Specific Symbiosis

**Figure 6:**
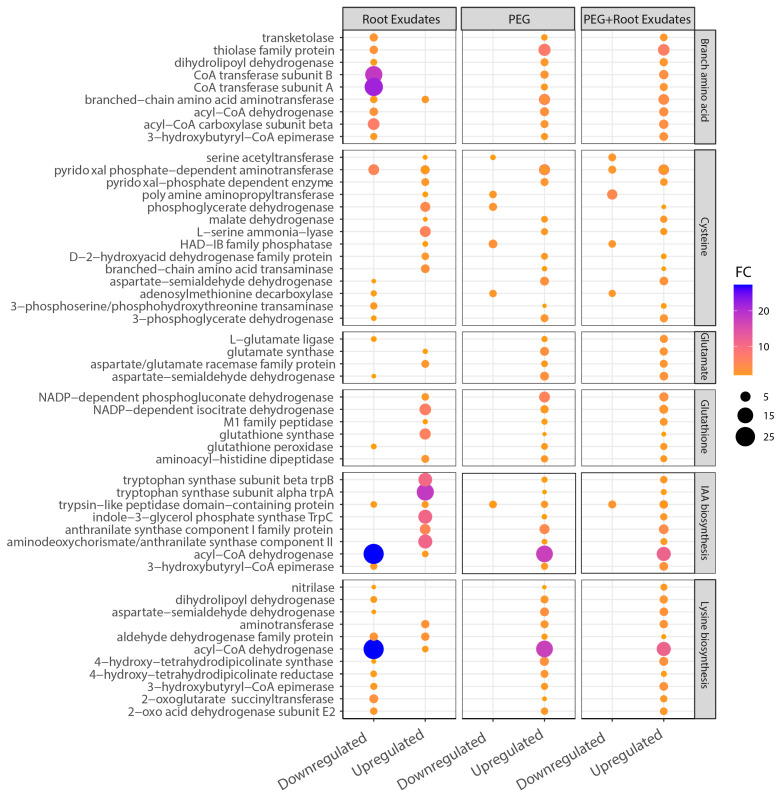
Functional enrichment analysis of highly regulated genes in *S. nripensae* DR205. Fold change of top up- and downregulated gene clusters related to biosynthesis and metabolism of branched-chain amino acids (valine, leucine, and isoleucine), cysteine, glutamate, glutathione, IAA biosynthesis, lysine biosynthesis in *S. nripensae* DR205 under treatments with root exudates, PEG, and PEG + root exudates. Fold changes represent gene expression in each treatment (root exudates, PEG, and PEG + root exudates) relative to untreated control cells cultured in M9 medium.

Among the diverse metabolic changes induced by drought, BCAA and lysine biosynthesis pathways emerged as the most dramatically regulated systems, showing opposite expression patterns between drought and non-drought conditions. This reversal in gene expression suggests that these pathways function as molecular switches for drought-activated symbiosis (Figure 6). Under root exudates alone, genes encoding BCAA biosynthesis enzymes were significantly downregulated. Most notably, acyl-CoA dehydrogenase and CoA transferase subunits A and B, which catalyze critical steps in leucine, isoleucine, and valine metabolism, showed dramatic 21- to 27-fold suppression compared to the control. Similarly, other BCAA pathway genes, including branched-chain amino acid aminotransferase, 3-hydroxybutyryl-CoA epimerase, and dihydrolipoyl dehydrogenase, were downregulated by 3- to 15-fold. This comprehensive suppression of BCAA metabolism under normal conditions suggests that these pathways are actively repressed during conventional plant–microbe interactions.

In contrast, the same BCAA biosynthesis genes were strongly upregulated under drought conditions. In both PEG and PEG + RE treatments, acyl-CoA dehydrogenase and CoA transferase subunits increased by 11- to 17-fold. The entire BCAA biosynthesis pathway showed coordinated activation, with most genes displaying three to nine-fold upregulation, specifically under drought stress. This drought-induced activation was maintained even in the presence of root exudates (PEG + RE treatment), indicating that drought signals override the suppressive effects of plant compounds on BCAA metabolism.

Lysine biosynthesis genes exhibited similar drought-specific activation patterns. Key enzymes in the lysine pathway, including aspartate-semialdehyde dehydrogenase, dihydrodipicolinate synthase, and 2-oxoglutarate succinyltransferase, were downregulated by three- to eight-fold under root exudates alone but upregulated by four- to six-fold under drought conditions. The L-lysine synthesis gene cluster showed particularly strong activation in PEG-containing treatments, suggesting coordinated regulation with BCAA pathways.

In contrast to BCAA and lysine pathways, glutamate and glutathione biosynthesis genes showed consistent upregulation across all treatments, though with varying magnitudes. Glutamate synthesis genes increased by three- to five-fold regardless of drought status, while glutathione pathway genes showed slightly higher expression (four- to six-fold) under drought conditions. This constitutive activation suggests that these pathways perform general stress response functions rather than playing drought-specific roles. The presence of root exudates in combination with drought (PEG + RE) revealed interesting modulation of gene expression patterns. While BCAA and lysine pathways remained strongly activated, their expression levels were slightly attenuated compared to PEG alone, suggesting that plant signals fine-tune the drought response. Conversely, sulfur metabolism genes showed significant downregulation (6- to 15-fold), specifically under the PEG + RE condition (Figure S3b).

These results identify BCAA and lysine biosynthesis as central metabolic hubs that undergo dramatic reprogramming in response to drought, reversing from strongly suppressed states under root exudates alone (21–27-fold down) to highly activated states under drought conditions (11–17-fold up). Importantly, this drought-induced activation was maintained at similar levels regardless of root exudate presence (PEG vs. PEG + RE), demonstrating that drought signals override root exudate-mediated suppression. This metabolic switch appears to be the key mechanism enabling DR205’s selective symbiotic behavior, providing both stress tolerance compounds for bacterial survival and potentially beneficial metabolites for drought-stressed plants.

## 4. Discussion

The present study reveals a previously unrecognized phenomenon in plant–microbe interactions, where *S. nripensae* DR205 exhibits drought-activated bacterial symbiosis through metabolic reprogramming centered on BCAA biosynthesis. This conditional mutualism challenges the conventional paradigm of PGPR functionality, where beneficial bacteria are generally expected to either function constitutively or show reduced activity under stress conditions. The discovery of a bacterium that specifically activates its plant growth-promoting capabilities only during drought stress represents a fundamental shift in understanding how beneficial microbes can adapt their symbiotic strategies to environmental conditions. This drought-specific activation mediated by a complete reversal in amino acid metabolism from suppression to strong upregulation suggests that some rhizobacteria have evolved conditional cooperation strategies that optimize resource allocation based on environmental stress signals and host plant needs.

The selective activation of plant growth promotion under drought represents an adaptive strategy distinct from known PGPR mechanisms. The suppression of BCAA biosynthesis during normal conditions followed by strong activation under drought indicates metabolic resource allocation based on environmental water availability. This pattern differs fundamentally from constitutive IAA producers such as some Pseudomonas strains that show less pronounced water-dependent regulation [15] and algal–bacterial systems where tryptophan-dependent IAA production via LAO mediates constitutive interactions [29]. In contrast, DR205 suppresses IAA biosynthesis genes under drought while activating BCAA and lysine pathways, representing a metabolic shift from auxin-mediated signaling to amino acid provisioning. This system also differs from *Bacillus* strains that primarily rely on sporulation under severe stress rather than maintaining active metabolic support [30]. The energy cost of BCAA biosynthesis provides context for this conditional activation. Bacteria tightly regulate BCAA synthesis through feedback mechanisms because their biosynthesis is metabolically expensive, requiring reducing equivalents and multiple enzymatic steps [31,32]. By linking BCAA production to drought sensing, DR205 invests metabolic resources only when there is a symbiotic benefit, potentially including access to plant-derived carbon compounds and root exudates in exchange for stress-alleviating amino acids. This metabolic cost–benefit balance aligns with fitness optimization models where facultative mutualists adjust cooperation intensity based on environmental context and partner availability [33]. The environmental origin of DR205 from drought-prone agricultural soils in Gangwon Province provides ecological context for this adaptation. In environments with periodic but not constant drought stress, bacteria that can switch between free-living and symbiotic lifestyles have competitive advantages over obligate symbionts or constitutive PGPR. The ability to sense and respond to water deficit allows rapid adaptation to changing conditions without maintaining costly biosynthetic machinery during favorable periods.

The coordinated upregulation of BCAA and lysine pathways under drought serves interconnected functions in bacterial stress tolerance and plant support. In bacteria, BCAAs contribute to osmotic adjustment through multiple mechanisms. BCAA biosynthesis provides precursors for branched-chain fatty acid (BCFA) synthesis, where valine, leucine, and isoleucine are converted to branched-chain acyl-CoAs that serve as primers for fatty acid synthase [34,35]. The resulting BCFAs increase membrane rigidity by introducing methyl branches that reduce membrane fluidity and water permeability [36], which is critical for maintaining cellular integrity under hyperosmotic conditions. When BCAA levels exceed biosynthetic needs, they can be catabolized through the branched-chain keto acid dehydrogenase complex to generate acetyl-CoA and propionyl-CoA, providing both energy and precursors for stress-responsive metabolites [37,38]. The upregulation of acyl-CoA dehydrogenase and CoA transferase subunits specifically under drought conditions connects BCAA metabolism to broader metabolic networks. These enzymes catalyze the oxidation of branched-chain acyl-CoAs generating FADH2 for energy production while producing intermediates that feed into the TCA cycle [39]. Similarly, enhanced lysine biosynthesis supports osmoprotection through its conversion to cadaverine by lysine decarboxylase, where cadaverine functions as a compatible solute that stabilizes proteins and membranes under osmotic stress [37]. These amino acid biosynthetic pathways are co-activated because they share nitrogen assimilation machinery and provide complementary stress protection through BCAA-mediated membrane remodeling and cadaverine-mediated macromolecular stabilization.

For plants, bacterial BCAA production during drought addresses specific metabolic demands. Drought stress in plants increases protein turnover and demands for stress-responsive protein synthesis [40,41]. BCAAs constitute approximately 20% of protein amino acids and are often limiting for protein synthesis under stress conditions [42,43]. Bacterial provision of these amino acids could support continued protein synthesis when plant BCAA biosynthesis is compromised by drought. Furthermore, leucine serves as a precursor for JA biosynthesis through the lipoxygenase pathway, with drought stress increasing JA accumulation for stomatal closure and stress gene activation [8]. The observed growth enhancement in DR205-inoculated plants under drought aligns with these metabolic support mechanisms. The specific reversal of BCAA and lysine pathways from suppression to activation uniquely under drought indicates specialized functions beyond general stress response. This selective regulation suggests that DR205 distinguishes between baseline stress responses and environment-specific metabolic programs. Notably, our transcriptomic analysis revealed that L-amino acid oxidase (LAO) genes did not exhibit significant differential expression under drought, root exudates, or combined conditions. This contrasts with algal–bacterial systems where LAO mediates tryptophan-dependent IAA production and auxin-regulated interactions [29]. The absence of LAO regulation in DR205 further supports the finding that amino acid biosynthesis, particularly BCAA and lysine pathways, constitutes the primary metabolic strategy for drought-activated plant support in this legume–bacteria system rather than amino acid catabolism or LAO-mediated auxin metabolism.

The suppression of BCAA genes by root exudates under normal conditions indicates that plant signals actively regulate bacterial metabolism. Root exudates contain diverse organic acids, sugars, and amino acids that can serve as both nutrients and signals for rhizobacteria [44,45]. The presence of exogenous amino acids from plants may trigger feedback inhibition of bacterial amino acid biosynthesis pathways through allosteric regulation of key enzymes such as acetohydroxy acid synthase, the first enzyme committed in BCAA biosynthesis [46,47]. This feedback mechanism would prevent wasteful biosynthesis when amino acids are available from the environment. Moreover, bacterial scavenging of nitrogen from plant-derived amino acids and peptides represents a nutritional benefit for DR205 under normal conditions, suggesting a bidirectional nutrient exchange that shifts under drought: from plant-to-bacteria nitrogen flow during favorable conditions to bacteria-to-plant amino acid provisioning during water deficit [48]. This metabolic reciprocity reinforces the conditional nature of the mutualism. Critically, transcriptomic profiles under treatment with PEG alone and PEG + root exudates were remarkably similar, demonstrating that drought signals override plant signals in activating BCAA and lysine biosynthesis. This drought signal dominance represents the mechanistic basis for conditional mutualism, ensuring that metabolic investment in plant support occurs specifically during water deficit when both bacterial survival and plant needs are greatest. This hierarchical regulation distinguishes drought-activated symbiosis from constitutive PGPR systems where plant signals alone trigger beneficial traits. The stable expression of secretion systems across conditions while biosynthetic pathways undergo dramatic changes indicates that export machinery remains constitutive, ensuring rapid metabolite delivery when production is activated.

Several aspects of the proposed mechanism require further investigation. Direct evidence for amino acid transfer from bacteria to plants through isotope labeling experiments would strengthen the symbiotic model. The specific osmosensing mechanisms and regulatory networks controlling the BCAA switch remain to be elucidated through genetic approaches. Additionally, the ecological relevance of this phenomenon in natural soil communities with complex microbial interactions needs field validation. The identification of BCAA metabolism as a key mediator of drought-specific symbiosis provides new insights for understanding and manipulating plant–microbe interactions. Future work should examine whether similar conditional mutualisms exist in other rhizobacteria from water-limited environments, potentially revealing convergent evolutionary strategies. Understanding the molecular dialog between drought-stressed plants and responsive bacteria could inform strategies for engineering enhanced stress tolerance in agricultural systems facing increasing water scarcity.

## 5. Conclusions

This study demonstrates that *Sphingobacterium nripensae* DR205 represents a new class of conditional mutualist that selectively enhances plant growth only under drought stress through dramatic metabolic reprogramming. The reversal in BCAA biosynthesis gene expression between normal and drought conditions reveals a metabolic switch mechanism unprecedented in the PGPR literature. This drought-activated symbiosis, mediated by the BCAA–lysine metabolic axis, provides dual benefits of bacterial stress tolerance and plant metabolic support precisely when needed most. The findings challenge the conventional view of plant-beneficial bacteria as constitutive helpers and instead reveal environmentally responsive cooperation strategies. Given the increasing impacts of global water scarcity and climate change on agriculture, understanding and harnessing such conditional mutualisms offers promising insights for developing climate-resilient crop production systems. Future development of DR205 and similar conditional PGPR could enable targeted microbial applications that activate specifically during drought events, providing efficient and sustainable solutions for maintaining crop productivity under water-limited conditions.

## Data Availability

The original contributions presented in this study are included in the article/Supplementary Materials. Further inquiries can be directed to the corresponding authors. All sequences from RNAseq data were deposited in the Sequence Read Archive of the NCBI under accession numbers (Bio project PRJNA895409; Bio sample SAMN31508455, SAMN31508456, SAMN31508457, and SAMN31508458).

## Supplementary Materials

The following supporting information can be downloaded at https://www.mdpi.com/article/10.3390/microorganisms14010114/s1, Table S1. RNA sequencing quality statistics and mapping rates for *S. nripensae* DR205 transcriptomic analysis. Table S2. Genomic features of *S. nripensae* DR205. Table S3. Genes incorporated in secondary metabolites by *S. nripensae* DR205. Figure S1. Genome circular map and Average Nucleotide Identity (ANI) of *S. nripensae* DR205. Figure S2. Transcriptomic modulation in *S. nripensae* DR205 genes in response to drought stress and plant–bacteria symbiosis. Figure S3. Gene expression analysis of highly regulated colonization functions and energy metabolism in *S. nripensae* DR205.

## Abbreviations

The following abbreviations are used in this manuscript:

PGPR	Plant growth-promoting rhizobacteria
BCAA	Branched-chain amino acid
NCBI	National center for biotechnology information
PEG	Polyethylene glycol
KEGG	Kyoto Encyclopedia of Genes and Genomes
GO	Gene Ontology
