# Supplementary material for: A Drought-Activated Bacterial Symbiont Enhances Legume Resilience Through Coordinated Amino Acid Metabolism
Source: Microorganisms. 2026 Jan 5;14(1):114. doi: 10.3390/microorganisms14010114 (PMC12844086; doi:10.3390/microorganisms14010114)
Supplement: Supplementary file 1 [file microorganisms-14-00114-s001.zip › microorganisms-4053174-supplementary.pdf]

Supplementary Information

A drought-activated bacterial symbiont enhances legume resilience through coordinated amino acid metabolism

Nishu Susmita Das[1], Jee Hyun No[1], Gui Nam Wee[1] and Tae Kwon Lee[1]*

[1]*Department of Environmental and Energy Engineering, Yonsei University, Wonju 26493, Republic of Korea*

*Corresponding author

Associate Professor, Department of Environmental Engineering, Yonsei University, Wonju 26493, Republic of Korea.

E-mail address: tklee@yonsei.ac.kr (T.K. Lee)

Phone: +82-33-760-2446

Fax: +82-33-760-5524

This file includes Table S1-S2 and Fig. S1, S2, S3

Table S1. RNA sequencing quality statistics and mapping rates for *S. nripensae* DR205 transcriptomic analysis

Treatment	Total read bases	Total reads	GC (%)	Q20 (%)	Q30 (%)	Processed reads	Mapped reads	Mapping rate (%)	Failed to align reads	Failed to align (%)	Suppressed reads by multiple mapping	Suppressed reads (%)
Control (1)	2,373,564,438	23,500,638	45.67	98.5	95.06	24,197,948	19,071,930	78.82	3,308,346	13.67	1,817,672	7.51
Control (2)	2,387,388,712	23,637,512	45.19	98.4	94.75	22,706,092	17,636,958	77.68	3,649,052	16.07	1,420,082	6.25
PEG (1)	2,135,105,660	21,139,660	44.76	98.4	94.7	24,289,250	18,483,580	76.1	4,220,284	17.38	1,585,386	6.53
PEG (2)	2,149,409,078	21,281,278	43.88	98.5	94.91	21,046,018	16,133,038	76.66	2,842,178	13.5	2,070,802	9.84
Root exudates (1)	2,094,297,418	20,735,618	44.66	98.4	94.78	27,199,644	22,423,542	82.44	2,575,060	9.47	2,201,042	8.09
Root exudates (2)	2,587,677,570	25,620,570	44.47	98.3	94.5	27,202,092	21,774,336	80.05	2,822,648	10.38	2,605,108	9.58
PEG+Root exudates (1)	2,371,821,582	23,483,382	45.19	98.4	94.77	27,787,254	21,410,306	77.05	4,633,556	16.68	1,743,392	6.27
PEG+Root exudates (2)	3,270,012,158	32,376,358	44.67	98.6	95.13	23,620,010	18,404,718	77.92	3,987,092	16.88	1,228,200	5.2

Table S2 Genomic features of *S. nripensae* DR205

Feature	
No of Chromosome	1
No of Plasmid	-
No of Reads	147,646
No of Contigs	1
Genome size (bp)	7,251,189
Average coverage (Depth)	136
GC contents %	40.5
rRNAs	21
tRNAs	86
ncRNAs	3
Pseudo genes	45
Protein coding gene (CDS)	5,956

Table S3 Genes incorporates in secondary metabolites by *S. nripensae* DR205

Secondary metabolites	Function
Bacteriocin	Peptidic toxins, inhibit the growth of other bacterial strain
Terpene	Pesticide, determine the smell of many plants and herbs
Lanthipeptide	Biological activities, from antimicrobial to antiallodynic
Fervenulin	Nematicidal activity
Siderophore	High-affinity iron-chelating compounds

a

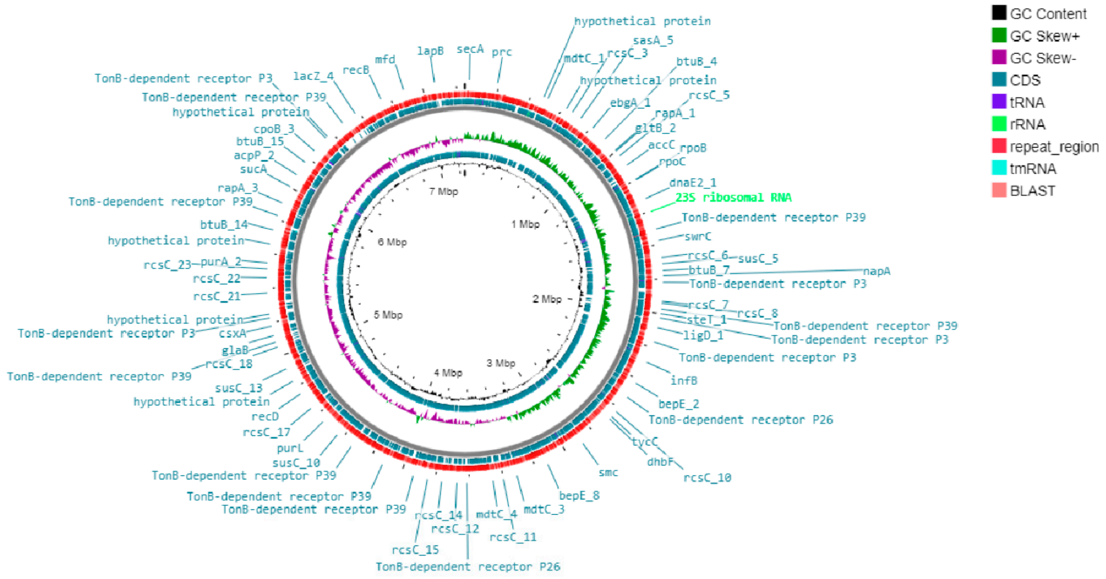

b

	S. nripensae DR205	S. pakistanesis B29	S. hotanense AMCC101218	S. lactis DSM22361	S. talpophilum NCTC11429
S. nripensae DR205	100				
S. pakistanesis B29	78.5	100			
S. hotanense AMCC101218	70.2	70.1	100		
S. lactis DSM22361	69.9	70.9	72.7	100	
S. talpophilum NCTC11429	77.2	76.2	69.8	70.0	100

Fig. S1. Genome circular map and Average Nucleotide Identity (ANI) of *S. nripensae* DR205. a. Circular representation of the DR205 genome. From the inside outwards, circle 1: scale; circles 2: GC content; circle 3 and 5: the predicted CDSs color-coded with tRNA, rRNA, repeat region, tmRNA; circle 4: GC skew (G+C/G−C) ; circle 6: BLAST annotated genes, respectively. b. ANI between the strain DR205 with other *Sphingobacterium* strains.

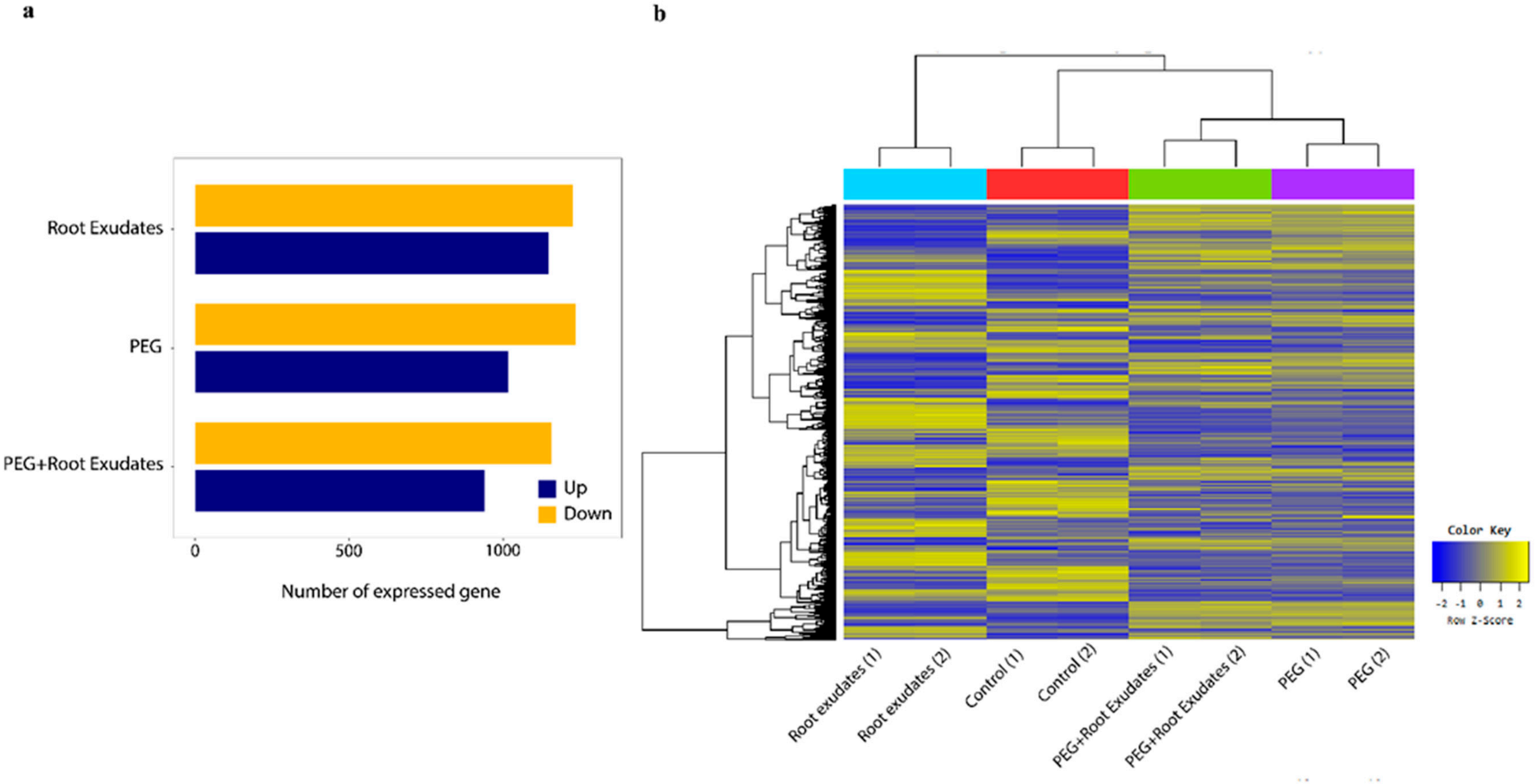

Fig. S2. Transcriptomic modulation in *S. nripensae* DR205 genes in response to drought stress and plant-bacteria symbiosis. a. Total number of up and down-regulated genes under the treatment of Root exudates, PEG and PEG+Root exudates. Yellow color indicates down-regulation and blue color indicates up-regulation of the genes. Gene regulation is compared to the gene expression without any treatment. b. Hierarchical Clustering and heatmap analysis for gene expression under the treatment of Root exudates, PEG, PEG+Root exudates, and control (no treatment) with two replicates.

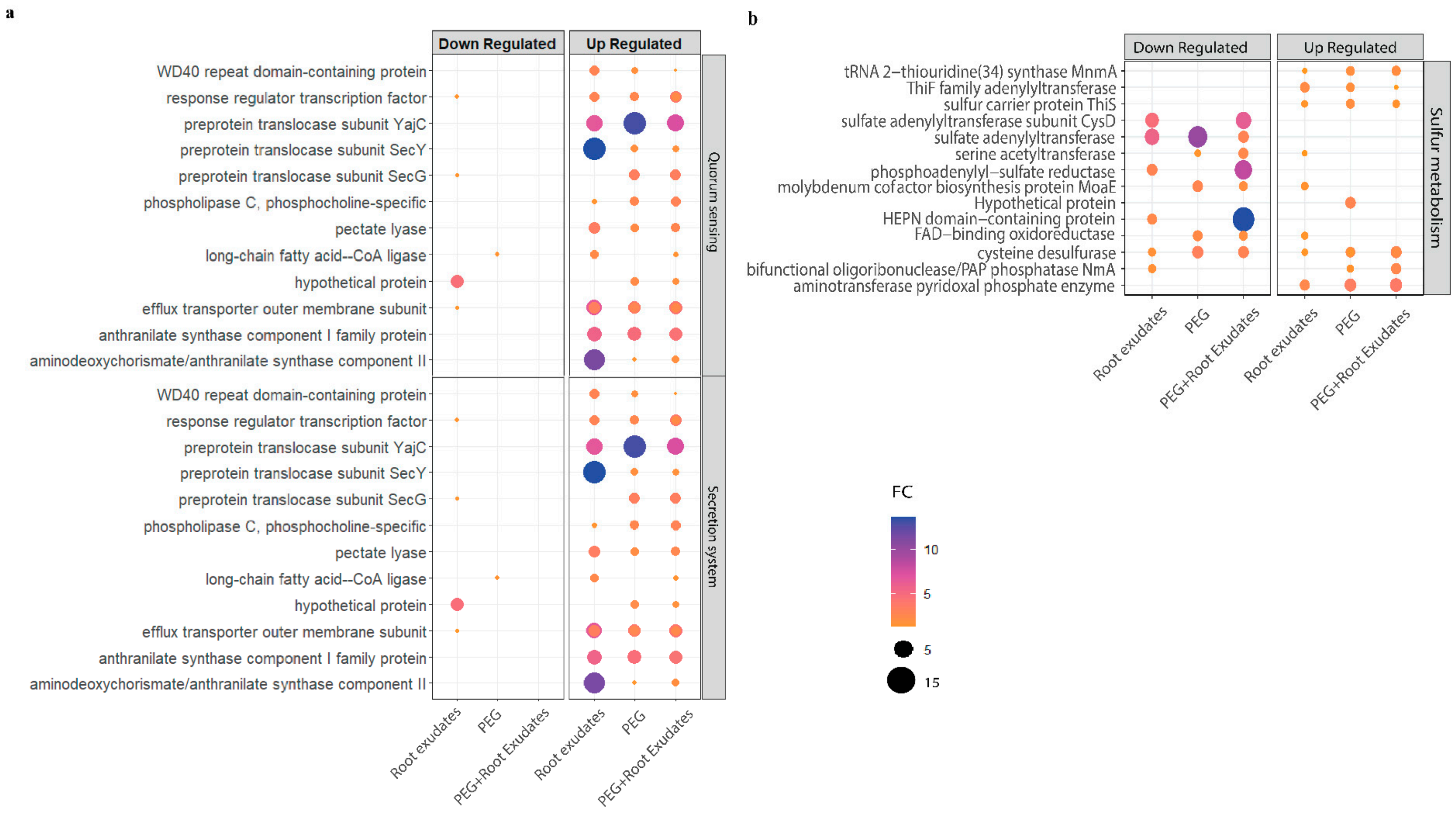

Fig. S3. Gene expression analysis of highly regulated colonization functions and energy metabolism in *S. nripensae* DR205. Fold change top up- and down-regulated gene clusters related a. Quorum sensing and Secretion system and b. Sulfur metabolisms as an energy source under the treatment of Root exudates, PEG and PEG+Root exudates.
